# Quality of life and care burden in primary caregivers of liver transplantation recipients in China

**DOI:** 10.1097/MD.0000000000010993

**Published:** 2018-06-15

**Authors:** Linbo Wei, Juan Li, Yanpei Cao, Jianming Xu, Wei Qin, Huijuan Lu

**Affiliations:** a School of Nursing; b Department of Nursing, Huashan Hospital; c Department of Nursing, Zhongshan Hospital, Fudan University, Shanghai, P.R. China.

**Keywords:** burden, caregiver, liver transplantation, quality of life

## Abstract

So far, liver transplantation (LT) has become the most effective way to treat end-stage liver-related diseases. As we all know, a stable caregiver is crucial to LT recovery. However, compared with LT patients, few studies were focused on LT caregivers.

This study aimed to describe the quality of life (QoL), care burden, and their related factors in caregivers of LT patients in Shanghai, China.

We collected 225 liver transplanted patients and their corresponding caregivers’ social demographic and medical information; meanwhile, we assessed 225 LT recipients’ caregivers’ life quality; by using Caregiver Quality of Life Index-Liver Transplantation in Chinese version (CQOLC-LT), Zarit caregiver burden inventory in Chinese version (ZBI) was used to access the care burden. Self-Rating Anxiety Scale (SAS) and Center for Epidemiological Survey-Depression Scale (CES-D) were used to assess caregivers’ anxiety and depression status. For physical assessment, we used Fatigue Scale-14 (FS-14). Logistic regression analysis was performed to identify clinical factors influencing QOL of LT primary caregivers.

The QoL of LT caregiver was not optimistic; the care burden on caregiver was still heavy. In particular, most caregivers experienced mild to moderate mental health disorders.

In general, our findings emphasized the importance of assessment of physical and mental health in primary caregivers during overall process.

## 1 Introduction

With the steady improvements in medical technique of organ transplantation and patients’ survival rate over the past 2 decades, liver transplantation (LT) has become an effective and optional therapy for many patients who have end-stage liver disease.^[1]^ It is undeniable that although LT can increase the patient's quality of life,^[2,3]^ it often causes chaos in the patients’ internal balance.^[4]^ In addition, in the post-transplant stage, patients’ survival rate depends on a lifelong immunosuppressant treatment. The LT recipients should follow strict medical and self-management program to avoid severe medical complications after LT, such as reaction of rejection.^[5]^ Therefore, a caregiver plays an important role in a transplant patient's health care during the whole recovery process. Actually, in most transplant centers, the LT recipients were requested to have a caregiver to provide care for them through the transplant process, as it can strengthen the information to patients and effectively help them in caregiving process, which could contribute to treatment.^[6]^

According to the research by Chen et al,^[4]^ caregivers offered company during medical treatment, food preparation, massage, daily assistance, medical care, and psychological support. LT is a very stressful process, which must be faced by the patients and their families.^[2,7]^ Thus, accepting the responsibility to take care of LT recipients is not an easy work, and many caregivers experience emotional challenges, for instance anxiety, depression, and caregiver burden.^[1,8]^ Several studies have shown that poor relatives’ status can place liver patients’ mental health and quality of life at risk.^[9]^ As such, acting as a primary caregiver becomes an ever-increasing challenge for family members.

Most studies in this field focused on LT recipients’ quality of life and mental health. Nevertheless, during this period, the primary caregivers were also influenced by providing emotional and day-to-day support to patients,^[10]^ and depression among primary caregivers is high.^[11,12]^ On the basis of these, the aim of our study was to explore the quality of life and care burden in primary caregivers and its related factors.

## 2 Materials and methods

### 2.1 Participants and procedure

A cross-sectional observational study was performed among outpatient and inpatient in LT primary caregivers from July 2014 to October 2015, with convenience sampling method from the Zhongshan Hospital of Fudan University, Huashan Hospital of Fudan University, and post-LT patient club in Shanghai, China. The sociodemographic and medical data of patients and caregivers were obtained. Then, inclusion and exclusion criteria were taken into consideration. The inclusion criteria were must being of adult age in both patients and caregivers; primary caregiver, which means family members who provided the major care and spent the longest time in daily care for patients; with no problem in understanding the evaluation instruments; and voluntary to participate in the study and sign the informed consent format. Caregivers with mental or cognitive disorders were excluded. On the basis of these criteria, initially, 240 LT caregivers were enrolled in this study; 15 subjects did not finish the questionnaire. At last, 225 LT primary caregivers were finally evaluated.

The Ethic Committee of School of Nursing, Fudan University approved the implementation of this investigation.

### 2.2 Instruments

The questionnaire was fulfilled by the subjects themselves and covered sociodemographic characteristics, which included relationship with patient, caregivers’ age, gender, occupation status, etc. We used Caregiver Quality of Life Index-Liver Transplantation (CQOLC-LT) in Chinese version to access LT caregivers’ QOL; this inventory consists of 35 items in 5 dimensions.^[13]^ Zarit caregiver burden inventory in Chinese version (ZBI)^[14]^ was used to access the care burden. The Chinese version of ZBI is a 22-item structured scale including 2 dimensions of personal strain and role strain. Other scales included Self-Rating Anxiety Scale (SAS) and Center for Epidemiological Survey-Depression Scale (CES-D) to evaluate mental health. For physical assessment, we used Fatigue Scale-14 (FS-14).

### 2.3 Statistics analysis

Database was established with Epidata 3.1 (The EpiData Association, Odense, Denmark), and data were analyzed using the SAS 22.0 (SAS Institute Inc. North Carolina University) statistic program. For parametric variables, differences between the 2 groups were analyzed using the independent samples *t* tests. One-way analysis of variance (ANOVA) was used to test the differences between more than 2 groups. Statistical relationships between variables were determined with Pearson correlation analysis. For example, the correlations of anxiety, depression, and social support with caregiver burden were identified using this method.

For nonparametric variables, the Mann–Whitney *U* test was applied for comparisons between 2 groups and the Kruskal–Wallis Test was performed to determine multigroup differences. Statistical relationships between variables were determined with Spearman correlation analysis. It was performed to identify whether caregiver burden was correlated with occupation and the relationship between caregiver and their patient. Multiple regression analysis was used for multivariate analysis. A *P* value of .05 was considered statistically significant.

## 3 Results

### 3.1 Description of primary caregiver and patient social demographics

Table 1 displayed the sociodemographic data of the primary caregivers of patients. Mean age of the caregivers was 51.71 ± 12.54 years, while mean age of patients was 56.43 ± 9.85 years. Among these 225 subjects, just over 60% caregivers were jobless. Roughly 50% patients and caregivers reflected they could cope with LT. There was a remarkable difference in gender distribution; caregivers were mainly female and patients were male, and most of them had husband–wife relationship, accounting for nearly 80% of them. Medical resources normally came from partial insurance; in other words, families paid medical expense partially by themselves, and the rest was paid by the government, which accounts for 58.2%. Over half of LT families bear sever economic burden (66.2%). Nearly half (46.2%) of patients had medical history, and most of them were hepatitis B. Most of the caregivers had no other caregivers to help them look after the LTRs; the percentage was 57.3%.

**Table 1:**
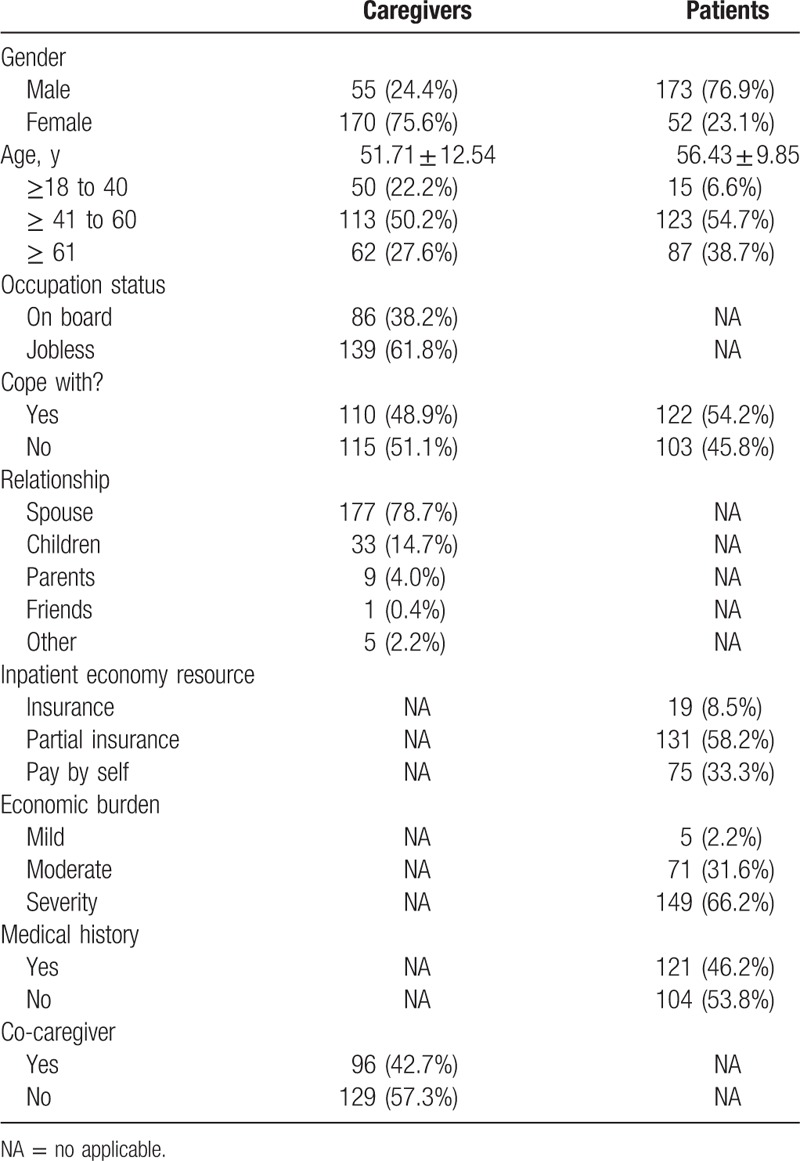
characteristics of LT patients and their primary caregiver.

### 3.2 Factors associated with primary caregiver's quality of life

Tables 2 and 3 described the factors associated with primary caregiver's quality of life. No matter for the caregivers or patients, coping with or not has a significant difference in the 2 groups (*P* = .007). For caring time last for 2 to 4 years, the QoL score was significantly lower than the other 2 groups (98.8 ± 15.08, *P* = .016). For those caregivers who care patients with medical history,QoL score was lower than that care patients without medicial history (*P* = .033). The caregivers had better score of QoL when medical cost was paid by insurance (112.7 ± 11.96, *P* = .039). In particular, having mild or moderate economic burden was associated with better QoL of caregivers in univariate analysis (*P* = .003), and patients who had more LT relative knowledge, their caregivers’ QoL scored higher than the other group (*P* = .023). Severity care burden, anxiety, and depression were associated with lower QoL score (*P* = .001); the results are reflected in Table 3.

**Table 2:**
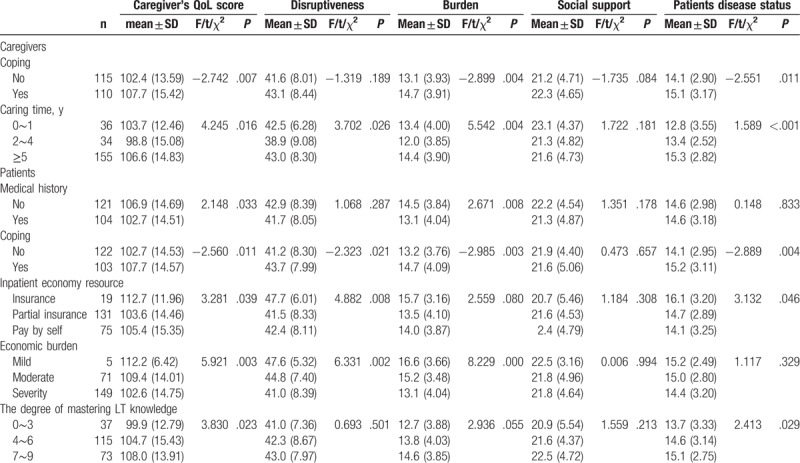
Social-demographic factors associated with primary caregiver's quality of life (n = 225).

**Table 3:**
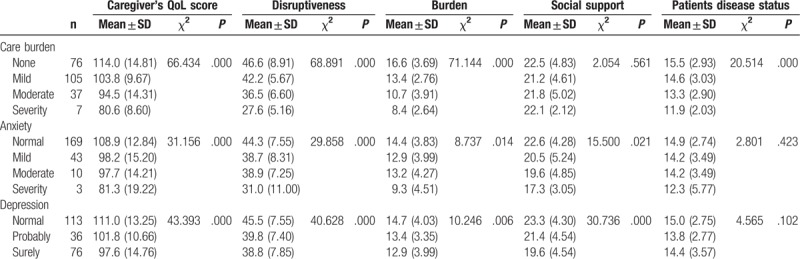
Psychosocial factors associated with primary caregiver's quality of life (n = 225).

### 3.3 Multiple regression analysis on the factors associated with quality of life

In the multiple regression analysis, LT families’ economic burden [standard partial regression coefficients (sb) = 1.777, *P* = .001], caregivers occupation (sb = 0.379, *P* = .002), medical history (sb = -5.059, *P* = .007), coping or not (sb = 1.878, *P* = .0029) were independent factors associated with QoL of caregivers (Table 4).

**Table 4:**
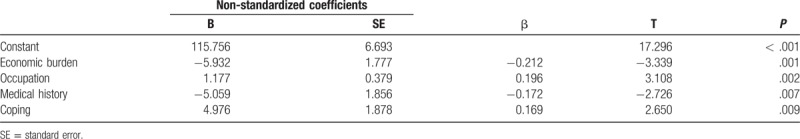
Multiple regression analysis on the factors associated with quality of life.

## 4 Discussion

The primary caregiver plays a critical role no matter in the pre- or post-LT process.^[12,15]^ The notable finding in this study is the heavy financial burden of medical-related expenses for LT patients; this situation happened particularly in families with lower income. The main reason is that the surgery itself is very expensive; meanwhile, the immunosuppressive agents are another expenditure after LT, due to the challenges from the tremendous pressure on follow-up medical care and ethical concerns.^[16]^ On the contrary, most patients’ primary caregivers in this project were middle aged. They bear heavy financial burden from taking care of the young and the old. For most of the time, primary caregivers have to suspend their own career to look after their loved ones, and this result was similar with the one by Chen et al.^[17]^ Nevertheless, losing financial resources probably increases the challenges that these caregivers face.^[18]^

Our results showed that patient's primary caregivers were mainly their spouse. This phenomenon maybe because of Chinese traditional views that female have the obligation to take over the caring tasks. The caregivers expressed their desire to participate in presentations and discussions on how to help their lover get through the ordeal. Our study also showed that caregivers who grasped more knowledge about LT would feel less anxious and depressed, as they knew how to handle daily tasks as well as some emergency situations. Moreover, knowing the information needs of caregivers is also essential to plan teaching-learning strategies to patients and families in liver transplant programs.^[19]^

LT has been described as a complex and sophisticated process with reduction of QOL of patients.^[20]^ Sometimes, patients still required early readmission after their initial hospital discharge.^[21]^ What should be demonstrated is that caregiver's impaired health status and/or poor quality of life can affect the quality of care that he or she provides to the patient.^[22]^ In this study, mean scores of caregivers was 14.73 ± 104.98, which means the quality of life of the caregiver was higher than predicted and the scores were similar with the original scale.^[23]^ This phenomenon happened maybe because the subjects in this study came from outpatient department, the patients were in a stable condition, and caregivers knew how to deal with daily tasks after LT.

Our study showed that caring time differences had a statistical significance among LTR caregivers. It is interesting to note that care time for 2 to 4 years got the lowest scores. Just as researchers reported that QoL of caregivers can be affected through neglecting their own needs for the benefit of patient over time.^[1]^

Consistent with previous study,^[24]^ our investigation also indicated that caregivers who suffered from severe care burden, dubious, and clinical levels of anxiety and depression had poor life quality. While there were also reported that caring transplant patients may bring benefits to caregivers, for instance caregivers may feel useful and needed, or they could discovery their inner strength from daliy caring, and at the same time developing a new life perspective.^[10,25]^

The average score for care burden in this study sample was 27.25 ± 15.12, which shows mild symptoms in this group. It should be stressed that those caregivers who bear heavy financial burden always have severe care burden. In addition, caregiver in husband–wife relationship scored the highest, which indicated that primary caregivers who are the spouse of the patients needed more support. In general, LTR should better have more than 1 caregiver; only in this way, other caregivers can assist primary caregiver for a short time, and then primary caregiver can take a rest, seek temporary respite and release their care burden to some degree.

Caring for a solid-organ transplant recipient may result in a greater feeling of loss of control than caring for patients with other diseases such as cancer; due to the postoperative condition, treatment of LT patients generally does not involve scheduled events.^[26]^ As reported above, a decline in caregiver mental health is especially important because caregivers may become unable to care for their chronically ill relative if significant burden and associated health impairments occur.^[18]^ And if the postoperation condition is not as expected, the primary caregiver can easily become a target of blame because he or she was the decision maker and the care provider.^[27]^ Our study indicated that nearly one half caregivers had mild to moderate anxiety and depression symptoms. Thus, early detection of caregiver distress and adjustment challenges may be helpful for both patients and caregivers.^[12]^ Professionals could offer caregivers timely support to help them overcome obstacles, such as encouraging them to participate in transplantation groups, to use more positive coping skills, even giving them access to these resources during the patients’ regularly scheduled clinic visits.^[24]^ These are effective ways in reducing psychological distress.

Another paper reported that anxiety and depression were related to waiting period for liver transplant, as the waiting time is a process of experiencing confinement,^[28]^ which should be taken into consideration.

It takes incredible investment of time and energy for caring LTR. Our findings, which are comparable to the results of caring for a chronically sick patient, may increase the risk of sleep disorder,^[29]^ thus leading to the feeling of tired.

Our study has some clinical implications. These results demonstrated the importance of physical and psychical intervention once the decision of doing LT is made, and should be both in patients and caregivers. In addition, according to our research, LT-related knowledge is rarely acquired from professionals, such as nurse. Nevertheless, till now, little information has been available for the organ transplant health professionals, especially for nurses in mainland China.^[17]^ It is also reported that nurses need to acknowledge that the patient's family members often have firsthand knowledge of patients’ preferences and can make an important contribution to their care.^[30]^ Findings of this project will raise clinical professionals attention to LTR families, thus motivating them to take specific measures to support LTR primary caregivers and should vary depending on different stages, at last, relieving caregivers’ care burden and stress and improving their life satisfaction.

In addition, this study has some strengthens that should be noted. In our study, we investigated the primary caregivers’ living status comprehensively. We used revised CQOLC-LT questionnaire to investigate the QoL of caregiver for the first time. Another aspect to be considered was that this study evaluated the largest number of caregivers of liver transplant recipients in China according to current researches, which means, our sample size was adequate, and at the same time, had good statistical power for analysis.

However, this study has limitations and should be considered in the further researches. This was a cross-sectional investigation, caregivers’ experience may change over time, although a paper reported that caregivers caregiving strain and mood disturbances are just as prominent in the months and years following LT as they were during the pre-LT waiting-period.^[15]^ Second, most of the subjects came from outpatient department, patients health status often tend to be stable, and we recommended that following research can add more inpatients. What is more, as this project was done in Shanghai, where the education level, economic, and living standards are higher than other districts in China, their experiences may not be identical to those having different backgrounds.

In general, the main contribution of this study is that we highlighted the necessities that health care professionals should pay more attention on LT caregivers’ physical and mental health, adopt effective measures to help them to face the challenges that occurred during LT recovery process, step by step, and improve their quality of life to the best.

## Author contributions

**Conceptualization:** Huijuan Lu.

**Data curation:** Linbo Wei.

**Formal analysis:** Wei Qin.

**Funding acquisition:** Huijuan Lu.

**Investigation:** Jianming Xu.

**Methodology:** Juan Li.

**Project administration:** Huijuan Lu.

**Resources:** Huijuan Lu.

**Software:** Yanpei Cao.

**Writing – original draft:** Linbo Wei.

**Writing – review & editing:** Huijuan Lu.
